# Synthesis and Characterisation of Bioactive Fluorescent FITC-Insulin Glulisine Conjugates for Potential Use in Insulin Delivery

**DOI:** 10.3390/pharmaceutics18030300

**Published:** 2026-02-27

**Authors:** Unmesh J. Desai, M. Joan Taylor, Sangeeta Tanna, Neill Horley, Mohsen Seifi, Raymond N. Allan, Michal Kozielecki, Harprit Singh, Dolgormaa Janchivlamdan, Joseph Festa, Andrew R. Bottrill, Ahmed Alsabih, Tarsem S. Sahota

**Affiliations:** 1 Leicester Institute for Pharmaceutical Innovation, Faculty of Health and Life Sciences, De Montfort University, The Gateway, Leicester LE1 9BH, UK; udesai01@dmu.ac.uk (U.J.D.); raymond.allan@dmu.ac.uk (R.N.A.);; 2 Allied Health Sciences, Faculty of Health and Life Sciences, De Montfort University, The Gateway, Leicester LE1 9BH, UK; harprit.singh@dmu.ac.uk (H.S.);; 3 Proteomics Research Technology Platform, School of Life Sciences, University of Warwick, Gibbet Hill Road, Coventry CV4 7AL, UK; 4 Department of Physiology, College of Medicine, King Saud University, Riyadh 11461, Saudi Arabia

**Keywords:** insulin, fluorescent labelling, FITC-labelled insulin, biologically active FITC-insulin, FITC

## Abstract

**Background/Objectives**: Drug development and delivery remain critical areas of research for addressing modern bioanalytical challenges. Understanding drug biodistribution, stability, and metabolism within biological systems is essential for optimising therapeutic efficacy. This study focuses on synthesising and characterising a novel fluorescent conjugate derived from commercially available rapid-acting insulin glulisine (Apidra^®^) and fluorescein isothiocyanate (FITC). The objective was to produce a mono-labelled FITC-insulin glulisine conjugate without employing complex protective group strategies or multi-step processes. **Methods**: The conjugation was optimised by varying molar ratios (1:1 to 3:1) and reaction times (18–24 h) at pH 7. **Results**: The desired B1 mono-labelled conjugate was successfully achieved at a 2:1 molar ratio, pH 7, and 18 h reaction time. MALDI-TOF mass spectrometry confirmed the molecular weight and conjugation site, with fragmentation analysis identifying FITC attachment at phenylalanine (B1) on the β-chain (*m*/*z* = 537.11). Western blots performed on C2C12 skeletal cell lysates stimulated with the FITC–insulin glulisine conjugate showed Akt and IRS-1 activity similar to that of cells treated with native commercial insulin glulisine. Confocal imaging also demonstrated translocation of GLUT4 in FITC–insulin glulisine conjugate-treated C2C12 cells similar to that of commercial native insulin glulisine. Octanol-water partitioning studies assessed the physicochemical properties of the conjugate. **Conclusions**: This approach demonstrates an efficient method for fluorescent labelling of insulin analogues, enabling future applications in imaging, biodistribution studies, and pharmacokinetic profiling.

## 1. Introduction

Fluorescent molecules, also known as fluorophores, are compounds that are capable of absorbing light at a specific wavelength and then re-emitting it at a longer wavelength [1]. These molecules play a fundamental role in pharmaceutical research, especially in drug delivery systems, due to their ability to provide real-time, non-invasive insights into the mechanisms of drug transport, biodistribution, and release [2,3]. They are quite stable, with long shelf lives, safe and relatively inexpensive whilst still displaying high detection sensitivity, as long as they are conjugated in the correct region [4]. When conjugating fluorescent molecules with proteins, recent reviews have found that they can be particularly useful for several reasons; they can be visualised and tracked in living cells or tissues, particularly in the study of cellular processes, protein localisation, and dynamics in real time [5,6].

Fluorophores conjugated to proteins have become essential tools for investigating cell signalling, enabling real-time visualisation of ligand–receptor interactions, receptor internalisation, intracellular trafficking, and metabolic responses [7,8,9]. Among these, fluorescently labelled insulin conjugates are particularly powerful because they allow direct tracking of insulin itself as it engages with its receptor and initiates downstream signalling cascades. The most widely studied fluorescent insulin derivatives include fluorescein-based labelled species designed to preserve biological activity, with the first detailed study performed by Hentz et al. [10], which focused on the synthesis of fluorescein isothiocyanate (FITC), C_21_H_11_NO_5_S (FITC) to human insulin. They discovered that in the labelling process of human insulin with FITC, A1, B1, and B29 residues are targeted. The substitution of positions has a significant impact on biological activity, providing insights into the main residues involved in insulin binding. Notably, only the FITC label at the B1 position maintains complete insulin biological activity, showing that the B1 position is not involved in the binding to the insulin receptor, while A1 and B29 positions exhibit a considerable reduction, approximately 10–100 times lower, in insulin activity. The least active species were found to be the tri-conjugated species at positions A1, B1, and B29. This highlights the significance of A1 and B29 in insulin to the insulin receptor binding [10,11].

Jacob et al. [12] conducted a more detailed characterisation of fluorescein isothiocyanate-labelled insulin (FITC–insulin), examining how different reaction conditions influence the number and position of FITC molecules attached to several commercial insulin formulations as well as bovine insulin, and how these modifications affect biological activity. Their study successfully synthesised mono-labelled FITC–insulin conjugates at either the A1 (Gly) or B1 (Phe) residues when reaction times were short (≤5 h); however, some unlabelled insulin always remained in the mixture. At extended reaction times (>45 h), all native insulin was consumed, and the product shifted toward di-labelled (A1B1) and tri-labelled (A1B1B29) conjugates, indicating that the extent of labelling increases with time. The presence of preservatives such as phenol or m-cresol reduced mono-labelled product formation by around 10% and increased di-labelled species, showing that formulation excipients significantly influence labelling outcomes. They also found that commercial FITC–insulin contained approximately three fluorescein labels per insulin molecule and was purified using size-exclusion chromatography to remove unconjugated dye, ensuring suitability for in vitro and in vivo studies, but was shown to be tri substituted, thereby having severely diminished bioactivity.

Other researchers have found that protecting groups play a crucial role in the synthesis of FITC–insulin conjugates. Liu et al. [13] developed a site-specific protection strategy using an N-trifluoroacetyl (Tfa) protecting group, introduced via ethyl trifluoroacetate, to selectively protectinsulin’s reactive amide groups while leaving one target amide available for FITC attachment. The Tfa group was stable in both aqueous and organic media but could be efficiently removed under mild basic conditions, enabling controlled, regioselective FITC labelling at A1, B1, or B29 without generating the complex multi-labelled mixtures typical of other FITC labelling methods. This multi-step approach allowed them to synthesise three distinct, well-defined FITC–insulin derivatives, overcoming longstanding problems of heterogeneity in FITC–insulin preparations. More sophisticated studies have focused on achieving site-selective conjugation to reduce interference with receptor binding. Alkhawaja et al. [14] demonstrated that labelling insulin at GlyA1 or PheB1 produces mono-labelled conjugates (GlyA1-FITC-insulin and PheB1-FITC-insulin) that retain biological activity in vivo, showing that careful control of labelling position can prevent steric disruption of insulin–receptor interactions. They demonstrated that mono-labelled conjugates which have been selectively labelled on the A1 insulin chain position maintain bioactivity similar to that of native insulin; however, work was conducted by protecting the B1 position with tert-butoxycarbonyl (BOC) anhydride, necessitating a multi-step conjugation process. The A1 labelling position was found by Hentz et al. [10] to have slightly less bioactivity than B1, although the difference was small.

Shah et al. [15] developed a rapid, low-cost, and reliable method for producing FITC-labelled human insulin, by manipulating reaction time and FITC:insulin molar ratio and produced distinct species—mono-, di- and tri-conjugated FITC–insulin—within less than 4 h. However, they were not able to exclusively produce the mono-labelled B1 conjugate in a single reaction and relied on size exclusion chromatography to separate each product in very low yields. The also showed that the degree of FITC conjugation significantly affects insulin permeability across Madin–Darby canine kidney (MDCK) epithelial monolayers. Mono-conjugated FITC–insulin displayed higher permeability than native insulin, and this was attributed to an increased partition coefficient, whereas tri-conjugated FITC–insulin exhibited lower permeability due to its increased molecular weight hindering transport. These findings demonstrate that fluorescent labelling can either enhance or impair insulin transport depending on conjugation density, highlighting the importance of controlled mono-labelling for bioanalytical and transport studies.

Vu et al.’s study [16] was the first to report a high-yield single-step synthetic process for the synthesis of biologically active mono-labelled B1 FITC–insulin utilising recombinant human insulin, and confirmed that this conjugate retained approximately 99.5% of native insulin’s activity in AKT phosphorylation assays and showed no significant differences in GLUT4 translocation responses compared to native insulin, further validating its use as a functional probe for insulin signalling research. The authors synthesised FITC–human insulin selectively labelled at the B1 position as a mono-conjugate (monoB1) which showed bioactivity similar to that of native human insulin.

Cyanine (Cy) dyes provide an important alternative to fluorescein, offering far-red emission, high photostability, and low cellular autofluorescence. Cy5-labelled insulin has been synthesised using azide-modified insulin, yielding GlyA1-Cy5 insulin that retains full in vivo biological efficacy [14]. This study demonstrates that far-red fluorophores can be successfully conjugated to insulin without compromising its pharmacological properties, making them suitable for imaging in tissues where red-shifted fluorescence significantly reduces background signal. These Cy5–insulin conjugates were also characterised using mass spectrometry and shown to maintain structural integrity under the conditions required for labelling, providing a platform for longer-wavelength imaging where FITC and Alexa Fluor 488 are suboptimal due to spectral overlap and autofluorescence.

Lochhead et al.’s study [17] did not synthesise FITC–insulin or focus on fluorescent labelling chemistry, but it did use fluorescently labelled insulin (including FITC–insulin) to investigate insulin distribution in the trigeminal nerve and brain following intranasal administration. They demonstrated that intranasally delivered insulin can enter the central nervous system via nose-to-brain pathways, and the use of fluorescent insulin probes allowed the authors to visualise and track insulin movement along trigeminal nerve routes and into brain tissue. This study provides a useful application of fluorescent insulin conjugates used to follow insulin penetration into neural pathways and to map CNS distribution following non-invasive administration.

### Insulin glulisine (Apidra^®^)

**Figure 1:**
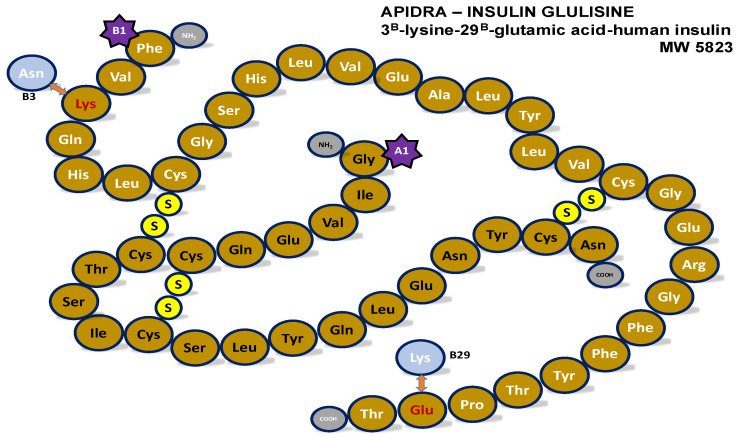
Structure of insulin glulisine with changes of amino acids at B3 and B29.

Insulin glulisine, commercially known as Apidra^®^, is an antidiabetic drug used to reduce high blood sugar and delivered by subcutaneous injection. It takes the form of a recombinant insulin analogue, modified to exhibit distinct characteristics as a rapid-acting insulin with a briefer duration of action compared to human insulin. It is predominantly monomeric, devoid of glycerol, and zinc-free, to prevent hexamer formation in solution form and thus facilitate rapid absorption. Maikawa et al. [18] stated, however, that removing zinc from insulin glulisine formulations by itself is insufficient to produce a monomeric insulin formulation, and hexamer formation by virtue of the cresol present in the formulation can occur, albeit to a lesser extent. The key difference between it and human insulin is the introduction of glutamic acid at position B29, as shown in Figure 1, replacing lysine found in human insulin [19,20,21]. Asparagine at B3 is also replaced by lysine.

In this study, a novel fluorescent insulin conjugate which is singly labelled at the B1 position in a one-step reaction under specific conditions will be shown utilising synthetic insulin glulisine which exists in predominantly monomeric form when in solution. This differs from other studies mentioned in that no protecting strategies are needed, as the reaction conditions which exploit FITC:insulin molar ratio and reaction time have been fine-tuned to yield the desired mono-labelled B1 (monoB1) conjugate in a single step with high yield. For comparative studies, i.e., between FITC–human insulin vs. FITC–insulin glulisine, this work will also utilise the monoB1 version of human insulin, which we have previously shown to be comparable to native human insulin in bioactivity [16], thereby showing a comparison for FITC–insulin glulisine with native insulin glulisine and also FITC–human insulin conjugate, which has been shown to be as bioactive as its native counterpart (i.e., human insulin). This provides a comparison further strengthening the work.

## 2. Materials and Methods

### 2.1. Chemicals and Reagents

Fluorescein 5-Isothiocyanate isomer I (FITC) (F-7250, CAS: 3326-32-7), C1C12 Skeletal Cells, and Sephadex^®^ G25 (G25150-50G, CAS: 9041-35-4) were acquired from Sigma Aldrich (St. Louis, MO, USA). The commercial insulins used were Apidra^®^ (insulin–glulisine, manufactured by Sanofi-Aventis, Paris, France) and Actrapid^®^ (recombinant human insulin) produced by Novo Nordisk (Bagsværd, Denmark), and were sourced from local pharmacies. The higher-grade chemicals were HPLC-grade acetone, acetonitrile (ACN), and trifluoroacetic acid (TFA). General laboratory-grade chemicals: Sodium dihydrogen phosphate (NaH_2_PO_4_), disodium hydrogen phosphate (Na_2_HPO_4_), glycerol, zinc oxide, n octanol, and m-cresol were all purchased from Fisher Chemicals (Loughborough, UK). Ethylenediamine tetra acetic acid disodium salt (EDTA) was purchased from Hopkins and William. Total AKT Mouse 1° Antibody, Mouse Phospho-IRS1 (Y612) 1° Antibody (tyrosine), Mouse Total IRS Antibody 1° Antibody, Anti-rabbit (IgG) secondary antibodies, Anti-mouse (IgG) secondary antibodies, Anti-goat (IgG) secondary antibodies, and Alexa Fluor 594 Secondary antibody were all purchased from R&D Systems (Abingdon, UK). GLUT4 primary antibody was acquired from Abcam (Cambridge, UK). Milli-Q water was used for the HPLC mobile phase and all relevant HPLC analysis and obtained from the Milli-Q UF Plus water purification system (Merck Millipore, Billerica, MA, USA). Double-distilled water was used for all the other solution preparations.

### 2.2. Synthesis of FITC–Insulin Glulisine Conjugate

The appropriate volume of FITC stock (25 mg in 1 mL of acetone) solution according to each molar ratio of FITC to insulin glulisine (Apidra^®^), namely, 3:1; 2:1 and 1:1, was added to 5 mL of Apidra^®^ solution (equivalent to 17.5 mg insulin glulisine) and adjusted to pH 7 to yield a bright orange solution for each conjugation. The mixture was left to react in the dark on a magnetic stirrer for the allocated reaction time at room temperature. The reaction time study was conducted at 18 and 24 h.

### 2.3. Purification of FITC–Insulin Conjugate by Gel Permeation Chromatography

Upon completion of the stipulated reaction time, the synthesised reaction mixture of FITC–insulin glulisine conjugate was injected onto a Gel Permeation Chromatography (GPC) assembly consisting of the following Buchii apparatus: chromatography pump B-688, peak detector B-686, fraction collector B-684, and a gel permeation column. The gel permeation column was composed of borosilicate clear plastic glass and packed with swollen slurry of Sephadex G-25 (bead size 50–150 µm). The entire synthesised reaction mixture, typically 5 mL of FITC–insulin glulisine, was injected onto the column and eluted with Milli-Q water. The first orange band of FITC–insulin glulisine was collected separately and lyophilised (Heto Drywinner, Wales, UK) to powder form after being snap-frozen in liquid nitrogen and stored at −20 °C until further analysis. The second and third bands containing unreacted FITC were discarded.

### 2.4. Reversed Phase High Performance Liquid Chromatography (HPLC) Analyses

The HPLC system (Shimadzu, Kyoto, Japan) used for the conjugate analysis consisted of the following components: In-line prominence degasser DGU-20AS, Prominence quaternary pump with four channels—LC-20AD, Prominence autosampler with 100 sample vial capacity—SIL-20A, Prominence column oven—CTO-20AC, Prominence diode array detector (DAD)—SPD-M20A, Prominence Fluorescence detector—RF-10AXL.

The column used for the HPLC separation of the components was a reversed phase octadecyl C18 Luna™ (3µ) column with dimensions of 150 mm length and 4.60 mm inner diameter. The stationary phase had a coating of 3 microns and was purchased from Phenomenex, Macclesfield, UK. The C18 column was preceded with a 0.5 mm in-line filter and a corresponding wide-pore C18, 4 × 3 mm guard column. The elution was achieved utilising a gradient method with a flow of 1.0 mL/min and a column being maintained at a constant temperature of 40 °C using the Prominence column oven—CTO-20AC and the sample injection of 50 µL loop. The gradient method used for the separation was 0–15 min (85% to 65% A), 15–25 min (65% to 35% A), and 25–45 min (35% A), where the mobile phase A was 0.1% trifluoroacetic acid (TFA) made up with Milli-Q water, and mobile phase B was 90% HPLC grade Acetonitrile and 10% of the 0.1% TFA used for mobile phase A.

A 50 µL sample injection was achieved using the SIL-20A Prominence autosampler. The peaks for the fluorescent detector were monitored using the optimum excitation and emission wavelengths for FITC and were achieved at 494 nm and 518 nm, respectively. The photodiode array detector was set to scan a range of 190–400 nm, and a specific channel was set at 276 nm to detect the unlabelled native commercial insulin glulisine.

The percentage AUP of chromatograms was calculated by area under a particular peak divided by the total area of all the peaks in a particular chromatogram multiplied by 100 and conducted using the HPLC software.

### 2.5. Mass Spectroscopy (MS) Analysis

The analysis of mass of the FITC–insulin glulisine conjugate was carried out by the Bruker ultrafleXtreme MALDI-TOF/TOF using Smartbeam-II™ laser, in linear and/or reflectron, positive and/or negative ion modes, with the mass range resolution of up to 40,000 (Billerica, MA, USA). Further analyses were carried out on the conjugate by nano electrospray-MS on the Orbitrap. The Advion Triversa Nanomate delivered the sample at a flow rate of 0.25 µL/min using a 96-well plate, utilising corresponding transfer tips and a 400-nozzle spray chip. The parameters set for the nano electrospray included spray voltage of 1.4 kV, gas pressure of 0.4 psi, transfer capillary temperature of 200 °C, and transfer capillary voltage of 30 V. Thermo Scientific LTQ Orbitrap^®^ XL was used as the Fourier-transformed frequency detector. The spectra were recorded at a resolution of 100,000 Full Width at Half Maximum (FWHM) over the *m*/*z* ranges of 150 to 2000 Da and 300 to 4000 Da with a mass accuracy of <3 ppm RMS.

### 2.6. MS Analysis of Labelling Position of FITC–Insulin Glulisine Conjugate

An MS Orbitrap Fusion with Ultimate 3000 RSL Cnano System from Thermo Scientific was used with the module consisting of TriVersa Nanomate nano spray source (Advion, New York, NY, USA) set to electrospray the samples directly using a flowrate of 300 nL/min, voltage of 1.4 kV, and gas pressure of 0.3 psi.

### 2.7. Cell Culture and Stimulation

The myoblast cell line C2C12 is an established cell model used to test insulin signalling molecules, including AKT, IRS-1, and GLUT4. Hence, these cells were used to examine the effects of commercial native insulin glulisine and its fluorescently labelled FITC–insulin glulisine conjugate. Cells were cultured using the medium DMEM containing 5% FBS at 37 °C in humidified 5% CO_2_. C2C12 cells were starved with serum-free media for 24 h and stimulated with or without the various insulin variants at 1 IU/mL at 37 °C for 15 min. Cells were then lysed using Laemmli Buffer.

### 2.8. Western Blotting and Immunofluorescence

Equal amounts of protein lysate samples were resolved on 10% SDS-PAGE and electro-transferred to a nitrocellulose membrane. Transferred proteins were then probed for phospho-AKT (pAKT), total AKT, Phospho IRS-1, and total IRS-1 using the appropriate antibodies. Immunoreactive proteins were visualised using chemiluminescent technique. For GLUT4 staining, C2C12 cells were stimulated as above and fixed with 4% paraformaldehyde, and then permeabilised with 0.5% TritonX-Tris-buffered saline. After blocking for non-specific binding using blocking solution (5% Milk powder and 95% TBST), cells were incubated with (1000×) GLUT4 primary antibody (ab654) for 90 min at room temperature. After washing with 0.1 M sodium phosphate buffer (pH7.4), cells were incubated with AlexaFluor 546 labelled secondary antibody (1:500) for 60 min before analysis using the EVOS^®^ FL Cell Imaging System (Thermo Fisher Scientific, Waltham, MA, USA).

### 2.9. Lipophilicity and Hydrophilicity of Conjugates

Saturated octanol and water were used where an equal amount of water and octanol was shaken and left for 24 h to reach saturation and then separated into two individual phases. A saturated octanol:water ratio of 2:1, at pH 7 was utilised with slow stirring to determine log P values for insulin glulisine and its corresponding conjugate. The aqueous layer was measured before and after 24 h at 276 nm, and concentration determined using a standard calibration curve of native and fluorescent insulin conjugate.

## 3. Results and Discussion

### 3.1. Effect of Molar Ratio and Reaction Time

**Figure 2:**
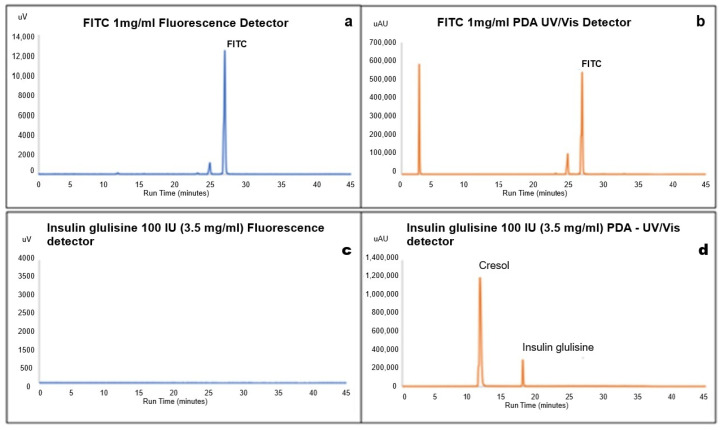
(**a**–**d**): (**a**) RP-HPLC fluorescence chromatogram (blue), (**b**) PDA UV–Vis (orange) of FITC (1 mg/mL) in acetone, (**c**) fluorescence chromatogram, and (**d**) PDA UV–Vis (orange) of insulin glulisine.

Figure 2a,b show fluorescence and UV chromatograms for neat FITC solution (1 mg/mL in acetone) with peaks at RT 24.5 and RT 26.85 min in both detectors.

Figure 2c shows the fluorescence detector chromatogram of insulin glulisine (100 IU, 3.5 mg/mL of insulin glulisine), where no peaks for insulin glulisine were observed, as it does not possess any fluorescent properties. The PDA UV–Vis chromatogram in Figure 2d shows the presence of insulin glulisine at a retention time of 18.96 min and m-cresol, the main excipient in the insulin glulisine formulation, at a retention time of 11.0 min. The presence of any of these peaks in subsequent chromatograms of the synthesised conjugated insulin glulisine demonstrates the presence of the undesirable unreacted FITC and unlabelled insulin post GPC purification.

**Figure 3:**
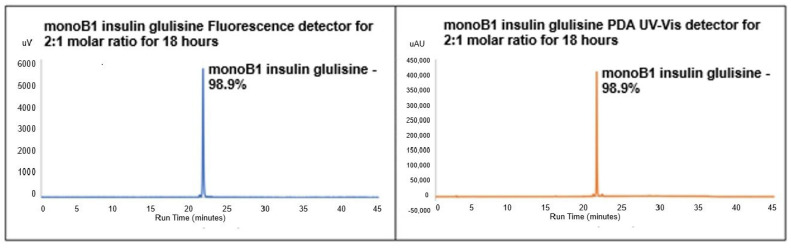
RP-HPLC fluorescence chromatogram and PDA UV–Vis of FITC–insulin glulisine conjugate synthesised at 2:1 molar ratio (FITC–insulin glulisine) after 18 h, showing a single mono peak at RT 20.97 min (98.9% AUP).

Previous studies by Vu et al. [16] using FITC–insulin (human) with a molar ratio of 2:1 and a reaction time of 18 h at pH 7 were considered to synthesise mono-labelled insulin glulisine conjugates. Figure 3 shows the results from fluorescence and PDA UV–Vis chromatograms, confirming the presence of predominantly mono-labelled (~99%) FITC–insulin glulisine conjugate in both detectors. The absence of any further peaks in both chromatograms shows that no other labelled conjugates (e.g., di- or tri-labelled) was present. It also shows the absence of unlabelled insulin glulisine, demonstrating the insulin glulisine under these reaction conditions had been solely mono-labelled. These HPLC results correlate to the previous FITC study with human insulin as opposed to insulin glulisine, which was also conducted at similar reactions conditions and yielded a mono-labelled peak at a similar retention time [16]. This is in contrast with the reports of [12] where 81% mono-labelled FITC–insulin glulisine conjugate was synthesized after 2 h, which decreased to 60% at 20 h when utilising a higher molar ratio of 3:1. They also yielded di-labelled conjugates of 9% and 40% at these respective rection times, which have been shown to be less bioactive.

Table 1 summarises the RP-HPLC analyses from syntheses carried out with FITC–insulin glulisine at molar ratios varying between 3:1, 2:1, and 1:1. Other than at the lowest molar ratio, the reaction time was fixed at 18 h. The overall yield of the desired mono-labelled FITC–insulin glulisine conjugate is expressed as a percentage AUP corresponding to each variation of the parameters. The di-labelled conjugate and the unlabelled insulin are also tabulated.

Table 1 shows the presence of substantial amounts of the di-labelled species produced when a molar ratio of 3:1 (FITC:insulin glulisine), reacting for 18 h at pH 7–7.5, with a reduced yield of 76% of the desired mono-labelled conjugate and 24% of the di-labelled with no unlabelled insulin detected in the PDA UV–Vis chromatogram. At a molar ratio of 1:1, the mono-labelled conjugate formed was 98% with 2% of the di-labelled; however, the PDA UV–Vis chromatogram confirmed the presence of 11% unlabelled insulin.

Further work was carried out to minimise the unlabelled insulin by increasing the reaction time to 24 h for a 1:1 molar ratio (pH 7), and more di-labelled species were produced compared to similar conditions at 18 h, with the amount of unlabelled insulin decreasing from 11% to 5%, indicating more di-labelled species (9%) conjugated product rather than the desired mono-labelled product.

### 3.2. Mass Spectrometry and Analytical Characterisation of the Mono-Labelled FITC–Insulin Glulisine Conjugate at the B1 Position

**Figure 4:**
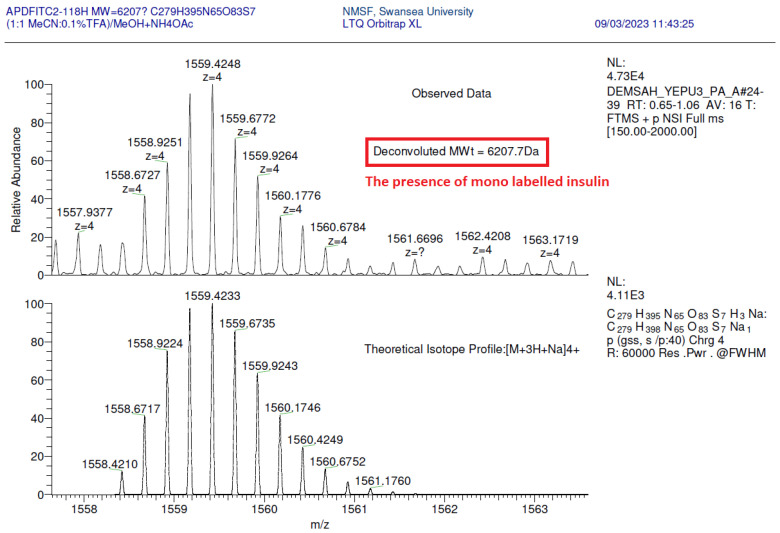
Mass spectrum data for the mono-labelled conjugate, confirming its molecular mass of 6207.7 Da.

**Figure 5:**
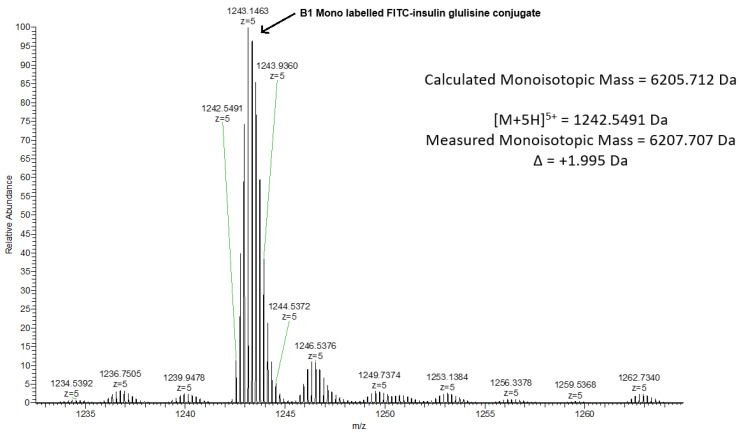
Monoisotopic data obtained from the mass spectra of the mono-labelled FITC–insulin glulisine conjugate at peak 1243.1463 with z = 5 charge.

Mass spectroscopic analysis was performed on the mono-labelled conjugate (C_279_H_395_N_65_O_63_S_7_) formed after 18 h when a molar ratio of 2:1 was utilised. Figure 4 and Figure 5 show that the majority of ions formed using the multiply charged ion yields are related to the proposed modified conjugate (envelope of 4+ ions around *m*/*z* 1553, with heterogeneity related to differential NH_4_^+^ and Na^+^ adduction at slightly higher *m*/*z*, deconvoluted to molecular weight of 6207.7 Da). This confirms that the insulin glulisine is conjugated with FITC at only one amino acid, hence confirming the mono-labelling.

The data obtained from the HPLC and MS analyses confirmed that the isolated mono conjugate was in its purest form, with no trace of the unlabelled insulin or the presence of any other FITC–insulin glulisine conjugates.

**Figure 6:**
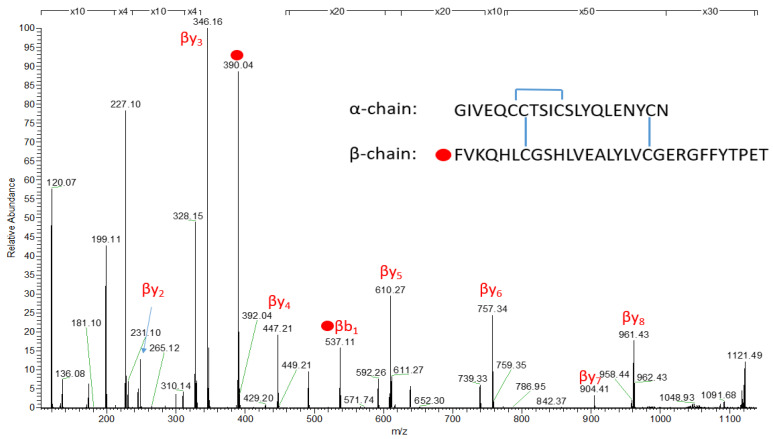
HCD MS2 fragmentation spectrum of 1243.1463 (5+) with red dots of fragments containing FITC. The *m*/*z* peak at 390.04 for the FITC was present at the first amino acid of the β-chain at position B1 (phenylalanine).

The conjugated sample was further analysed using MS to ascertain the labelling position of FITC. The amino acid phenylalanine on the β-chain at the B1 position of the insulin glulisine molecule was obtained by the HCD MS2 fragmentation spectrum of 1243.1463 (5+) and is shown in Figure 6 with red dots depicting the presence and position of the FITC (MW = 390.04) on the amino acid chain at B1 of the β-chain of insulin glulisine on phenylalanine (peak at 537.11).

### 3.3. Activity of Fluorescently Labelled Conjugates in C2C12 Cells

**Figure 7:**
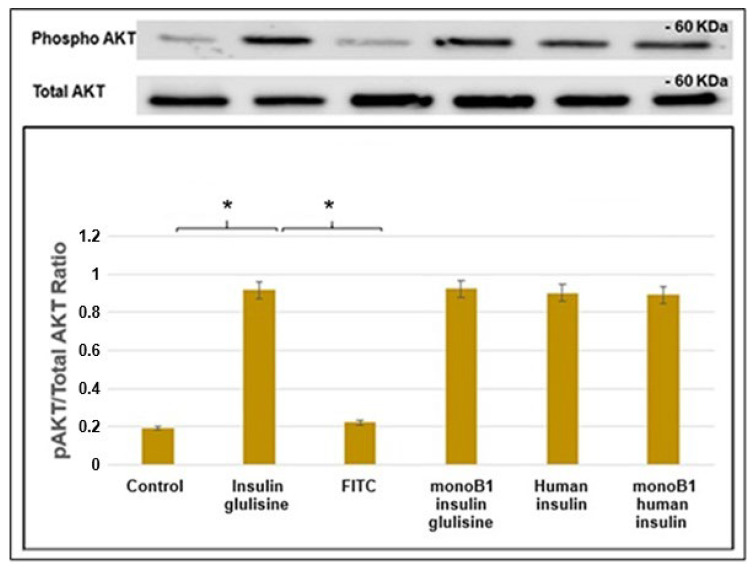
Effect of insulin, insulin glulisine, and fluorescently labelled conjugates on AKT phosphorylation in C2C12 cells. C2C12 cells were treated with the various insulin variants for 15 min before protein lysates were collected. Phospho-AKT and AKT were detected in whole cell lysates by immunoblotting. The ratio of phospho-AKT (pAKT) to total AKT was quantified across three independent experiments by scanning of relevant bands on blots. A significant difference was observed between the controls and the commercial insulins, with their conjugates indicated by * *p* < 0.05, Student’s *t*-test.

The biological activity of the FITC–insulin glulisine conjugate was determined by measuring serine phosphorylation of AKT (pAKT) where insulin stimulates the PI3K/AKT signalling pathway via insulin receptor substrate 1 (IRS-1) activation. Insulin-induced serine phosphorylation of AKT is a key molecular marker of insulin activity, which subsequently leads to GLUT4 expression and translocation [22]. For comparison, human insulin and previously synthesised monoB1 human conjugate [16] were also included. An increase in pAKT was observed in C2C12 cells stimulated with human insulin, insulin glulisine, and the corresponding mono conjugates compared to the control and FITC dye-treated cells (Figure 7). There was no significant difference in AKT activity between the commercial and conjugate insulin-treated cells.

**Figure 8:**
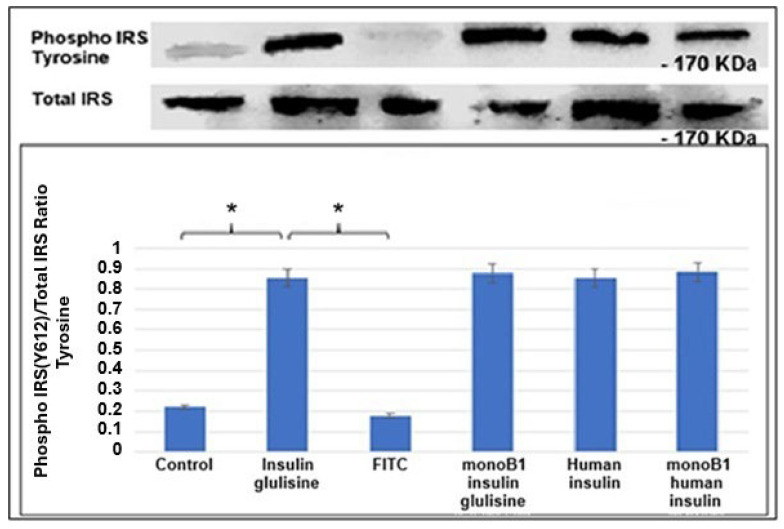
Effect of insulin and fluorescently labelled conjugates on IRS-1 phosphorylation. C2C12 cells were treated with the various insulin variants for 15 min before protein lysates were collected. Phospho-IRS1 and total IRS1 were detected in whole cell lysates by immunoblotting. The ratio of pIRS1 to total IRS1 was quantified across three independent experiments by scanning of relevant bands on blots. A significant difference was observed between the controls and the commercial insulins, with their conjugates indicated by * *p* < 0.05, Student’s *t*-test.

The effects of insulin and fluorescently labelled conjugates on the tyrosine phosphorylation of -IRS-1 (pIRS-1) in C2C12 cells were also examined. Insulin-induced tyrosine phosphorylation of IRS-1 is upstream of AKT activation, and so it was of interest to see if these levels correlated with pAKT. Similar levels of activity of IRS-1 were observed in all the different insulin variants tested, with all showing significant increases in pIRS-1 compared to controls (Figure 8).

**Figure 9:**
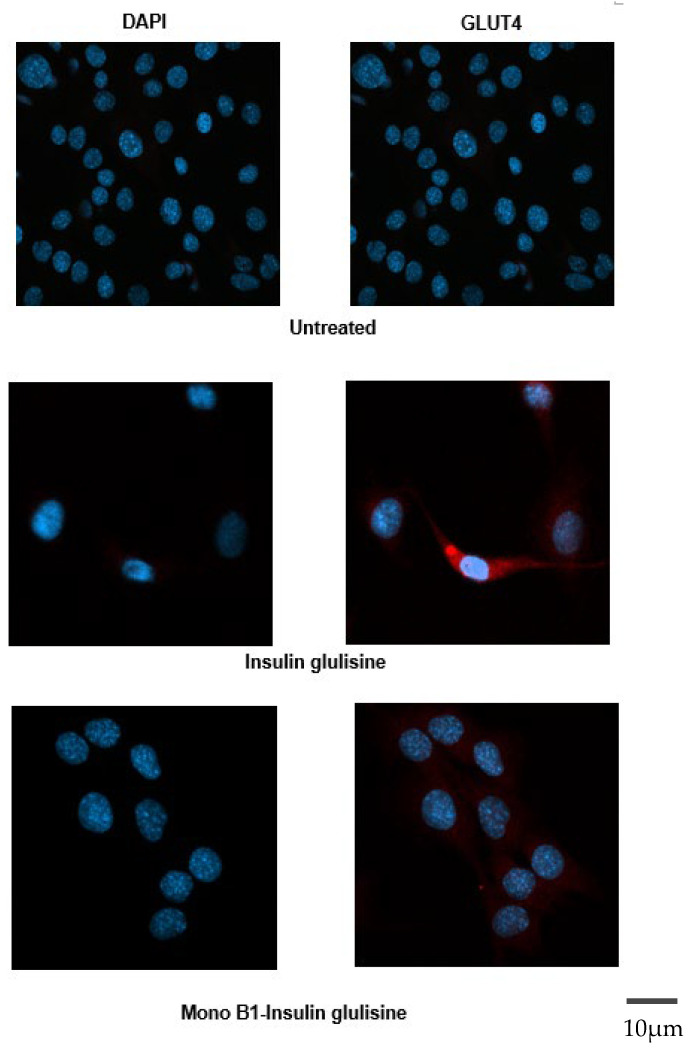
Effects of insulin glulisine and its conjugates on GLUT4 expression in C2C12 cells. Cells were treated with or without insulin glulisine conjugates for 15 min and fixed. Cells were then fluorescently stained for DAPI (blue) and GLUT4 (red).

To further confirm the biological activity, C2C12 skeletal muscle cells were stimulated with insulin glulisine and mono B1 labelled insulin glulisine, and GLUT4 expression was examined using immunofluorescence. Increased expression of GLUT4 was detected in insulin variant-treated cells compared to control. While the intensity of GLUT4 expression was observed more in the non-labelled insulin variant, the staining pattern was similar for both types (Figure 9).

### 3.4. Partition Coefficient (Log P) Analysis of Insulin Glulisine and Its FITC Conjugate

The slow-stirring method with saturated solvents was employed to determine the partition coefficient (log P) of insulin glulisine and its FITC-conjugated derivative. Using 500 ppm of insulin glulisine, the log P value was calculated as −1.95, consistent with previously reported values [23]. This strongly negative log P indicates high hydrophilicity, suggesting that insulin glulisine is more soluble in aqueous environments than in nonpolar solvents. Consequently, its low affinity for lipid membranes limits oral absorption due to poor intestinal permeability.

In contrast, fluorescein isothiocyanate (FITC) exhibits a log P of 5.25, reflecting pronounced lipophilicity [24]. Conjugation of FITC to insulin glulisine significantly alters the physicochemical profile of the molecule. At 50 ppm, the FITC–insulin glulisine conjugate displayed a log P of −0.197, indicating a substantial shift toward lipophilicity compared to native insulin. This modification is attributed to fluorescein’s weakly acidic nature and limited water solubility, which enhances the conjugate’s affinity for lipid environments.

The increase in lipophilicity may facilitate improved cellular uptake and distribution within adipose tissue following subcutaneous administration. Importantly, this conjugation did not compromise the insulin analogue’s ability to interact with its receptor, as shown previously. Incorporating a lipophilic moiety such as FITC into insulin analogues represents a promising strategy for developing advanced delivery systems that maintain therapeutic efficacy similar to that of their native counterparts and broadens the scope for potential modes of administration.

## 4. Conclusions

A streamlined and efficient protocol for FITC labelling of insulin glulisine has been established under mild conditions (pH 7, 2:1 FITC-to-insulin glulisine molar ratio, 18 h reaction time), achieving near complete yield without the need for protecting groups or multi-step synthesis. The resulting mono-labelled insulin glulisine (monoB1) retains full biological activity, including effective GLUT4 translocation via the insulin signalling pathway, comparable to native insulin. This approach offers a robust and simplified method for generating fluorescently conjugated insulin glulisine suitable for diverse biological and pharmaceutical applications, particularly in insulin delivery research.

## Figures and Tables

**Table 1:** HPLC AUP results from different reaction conditions for the synthesis of FITC–insulin glulisine conjugates.

Molar Ratio (FITC:Insulin-Glulisine)	Reaction Time (Hours)	Mono-Labelled Conjugate	Di-Labelled Conjugate	Unlabelled Insulin
3:1	18	76.0%	24.0%	none
1:1	18	98.0%	2.0%	11.0%
1:1	24	91.0%	9.0%	5.0%
2:1	18	99.0%	negligible	none

## Data Availability

The original contributions presented in this study are included in the article. Further inquiries can be directed to the corresponding author.
